# Mechanical properties of concrete filled steel tube members constrained by carbon fiber reinforced materials under bidirectional coupled loading

**DOI:** 10.1016/j.heliyon.2024.e34144

**Published:** 2024-07-04

**Authors:** Qing-li Wang, Hai-yu Qin, Kuan Peng

**Affiliations:** a School of Civil Engineering, University of Science and Technology Liaoning, Anshan, 114051, PR China; b School of Intelligent Manufacturing, Chengdu Technological University, Chengdu, 610031, PR China

**Keywords:** CFRP, CFST, Loading performance, Finite element analysis

## Abstract

Carbon fiber reinforced composite structures have been a research hotspot in recent years, with 9 specimens designed for static tests under bending and torsion loads of CFRP concrete filled steel tubes. The torque angle (*T*-*θ*) curve was studied from an experimental perspective. Subsequently, a reasonable finite element model was established using ABAQUS software. In addition, the effects of changes in parameters such as the number of steel concrete strength, bending ratio, and steel ratio on C–CF–CFRP-ST bending and torsion specimens were studied through numerical parameter research. Finally, equations of bearing capacity of CFRP-concrete filled steel tube under coupled bending and torsion are proposed.

## 1 Introduction

In recent years, concrete filled-steel tube structures have been increasingly widely used in high-rise buildings, industrial plants, and bridge structures [1,2]. As a new type of composite structure, concrete filled-steel tube has attracted attention from the civil engineering community both domestically and internationally due to its superior performance. From the perspective of mechanical properties, concrete filled-steel tube has high bearing capacity, good plasticity and toughness [[3], [4], [5], [6]]. When steel tube and concrete are subjected to pressure together, they can fully utilize the tensile performance of steel tube and compressive performance of concrete. Steel tube confined concrete not only improves the strength of concrete, but also enhances its plasticity and toughness performance, From a construction perspective, steel tubes can serve as construction templates, accelerating construction progress and saving construction costs. However, any structural form has its shortcomings. When the steel tube concrete column bears a large load, the corresponding component section must also be relatively large, which requires thicker steel tubes to provide sufficient clamping force to the concrete.Tightly adhere CFRP to the outer surface of steel tube concrete to form a new composite structure: CFRP concrete filled-steel tube. This new type of structure has advantages such as high bearing capacity, good durability, can be used to repair/reinforce existing steel tube concrete, delay the buckling of steel tubes, and superior ductility to FRP tube concrete [[7], [8], [9], [10], [11], [12], [13], [14], [15], [16]]. Moreover, this new structure has many other advantages over steel tube concrete, such as reducing the size of steel tube concrete, reducing its steel usage, and reducing the weight of components [17,18].

Ding et al. [19] conducted studies on restraint coefficient of the stirrups-stiffened square concrete filled double-skin steel tube axial compression stub columns. Peng [20] conducted a tensile test on 32 stainless steel tube concrete specimens. The main parameters of the test were void ratio and eccentricity. The study showed that void defects had little effect on the ultimate tensile bearing capacity, while eccentricity had a significant impact on the ultimate tensile bearing capacity. Hao H et al. [21] conducted axial tensile and flexural mechanical performance tests on elliptical section steel tube concrete, analyzed the influence of different loading points on the bearing capacity of the specimen and the stress state of different characteristic points of the specimen, and found that the steel tube has a greater constraint force on the concrete at the circular arc. Lin T [22] conducted research on common spherical crown void defects in engineering and completed tensile and bending tests on four stainless steel tube concrete with spherical crown void defects and four stainless steel tube concrete without void defects. Lian Q et al. [23] conducted axial tensile tests on 16 reinforced steel tube concrete specimens, studied the bond slip performance of reinforced steel tube concrete, explored the bond slip constitutive relationship between steel tube and concrete, and proposed a constitutive relationship model between steel tube and concrete under different circumferential pressures based on the test results. Guo H [24] conducted tensile loading tests on 22 specimens (including axial tension and bending), and obtained the failure mode, load deformation relationship, bearing capacity, and development of steel tube strain of stainless steel tube concrete (CFSST) tensile specimens. However, there is relatively little research on its shear performance.

In view of this, a testing device and method for structural members under bending-torsion have been developed; Conduct relevant experimental research with bending moment ratio and CFRP layers as the main parameters to investigate the influence of these parameters on the bearing capacity and stiffness of the specimen. Proposing the bending-torsion correlation equation for concrete filled CFRP steel tube members.

## 2 Experimental study

### 2.1 Specimens design and materials

#### 2.1.1 Specimens design

9 circular CFRP concrete filled steel tubular specimens were prepared. The main parameters include bending moment ratio (*m*). The bending moment is shown in (1), (2), (3), (4), (5), (6).

(1) $$m=M/M_{u}$$

(2) $$M=\frac{PL}{6}$$

(3) $$M_{u}=\gamma_{m}W_{\text{cfscm}}f_{\text{cfscy}}$$

(4) $$\gamma_{m}=\gamma +\left(0.3+0.2\xi \;\right)\eta_{\text{cf}}$$

(5) $$W_{\text{cfscm}}=\pi D_{s}^{3}/32$$

(6) $$f_{\text{cfscy}}=\left[1.14+1.02\left(\xi_{s}+3\xi_{\text{cf}}\right)\right]f_{\text{ck}。。}$$

where: *M* is the bending moment of the middle section of the member, *P* is the applied lateral force of the middle section, *L* is the length of the specimen, *M*_u_ is the flexural capacity of the member, *W*_cfscm_ is the flexural modulus of the member, *γ* and *γ*_m_ are the calculation coefficients of flexural capacity, *ξ* is the total restraint coefficient, *η*_cf_ is the longitudinal CFRP strengthening coefficient, *D*_s_ is the outer diameter of the steel tube, *f*_cfscy_ is the axial compression strength of the specimen, and *ξ*_s_ is the restraint coefficient of the steel tube, *ξ*_cf_ is the transverse CFRP restraint coefficient, and *f*_ck_ is the standard value of axial compressive strength of concrete.

**Fig. 1:**
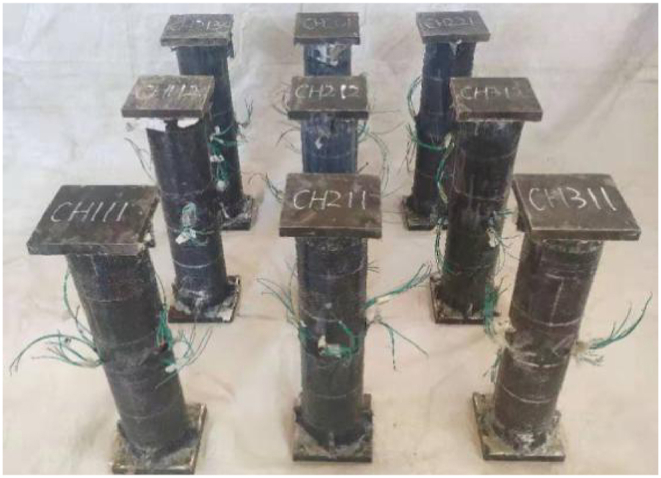
Concrete filled CFRP steel tube specimen.

In the test, *L* of all specimens is 540 mm, *D*_s_ is 120 mm, and the wall thickness *t*_s_ of steel tube is 3 mm. Other parameters are shown in Table 1. The prepared components are shown in Fig. 1.

**Table 1:** Parameters of C–CF–CFRP- ST specimens.

No.	Specimens label	*m*	*m*_l_/layers	*m*_t_/layers	*ξ*_cf_	*ξ*
1	CFT111	0.1	1	1	0.15	1.12
2	CFT211	0.2	1	1	0.15	1.12
3	CFT311	0.3	1	1	0.15	1.12
4	CFT112	0.1	1	2	0.31	1.28
5	CFT212	0.2	1	2	0.31	1.28
6	CFT312	0.3	1	2	0.31	1.28
7	CFT213	0.2	1	3	0.46	1.43
8	CFT201	0.2	0	1	0.15	1.12
9	CFT221	0.2	2	1	0.15	1.12

#### 2.1.2 Material properties

Steel tube of specimen are measured through test. The indicators of steel are measured by tensile tests, and the tensile test of steel is carried out according to Metallic materials-Tensile testing-Part 1: Method of test at room temperature (GB/T 228.1–2010). The indexes of steel obtained through specific tests are shown in Table 2.

**Table 2:** Properties of steel tube.

*f*_y_/MPa	*f*_u_/MPa	*E*_s_/GPa	*v*_s_	*ε* '/%
291.78	456.49	210.16	0.31	27.7

The indicators of concrete are measured by National Standard of the People's Republic of China. Code for Design of Concrete Structures(GB 50010-2010) The elastic modulus *E*_c_, concrete cube compressive strength *f*_cu_ are shown in Table 3.

**Table 3:** Properties of concrete.

*E*_c_/MPa	*f*_cu_/MPa	*f* '_c_/MPa
32500	48.89	37.62

The thickness of CFRP, elastic modulus *E*_cf_, transverse fracture strain *ε*_cftr_ and longitudinal fracture strain *ε*_cflr_ used for specimens are shown in Table 4.

**Table 4:** Performance of CFRP used for CFRP concrete filled steel tube specimens.

Thickness/mm	*E*_cf_/GPa	*ε*_cftr /_με	*ε*_cflr_/με
0.111	230	3000	5000

### 2.2 Loading and measurement

**Fig. 2:**
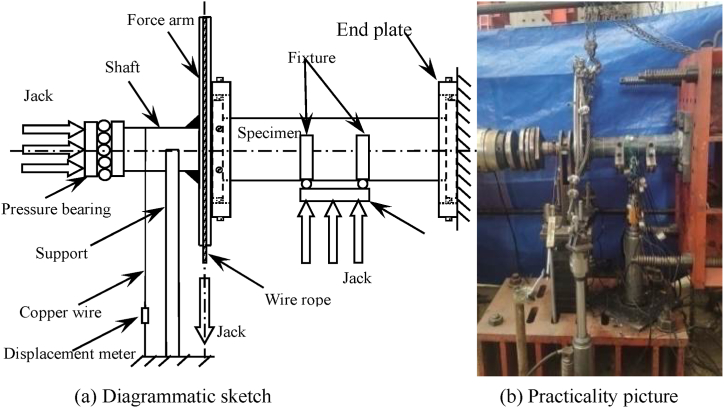
Loading equipment diagram of specimen.

Before the test, one end of the specimen was fixed on the embedded device as a fixed end, and the other end was fixed on the rigid arm. Connect one side of the rigid arm to the 20t jack with steel wire rope, and drive the rigid arm to rotate through the lifting of the jack, so as to apply torque. Two 20t jacks are fixed at the lower part of the test piece to exert the bending moment on the test piece. The loading device is shown in Fig. 2. Fig. 2(a) is diagrammatic sketch, and Fig. 2(b) is Practicality picture of equipment.

**Fig. 3:**
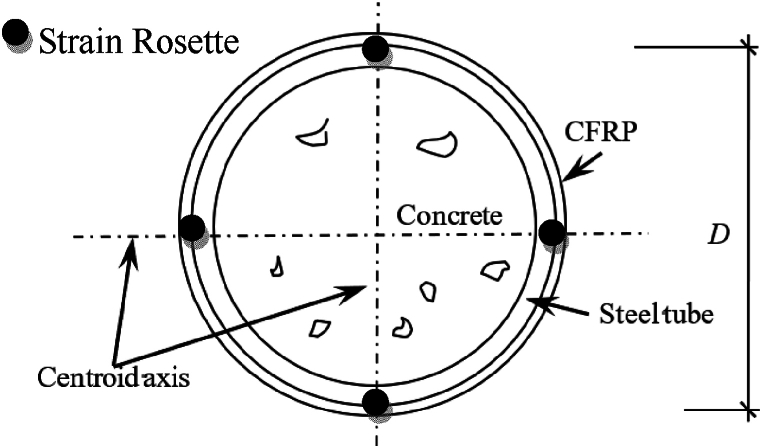
Arrange of strain rosette of specimen.

The estimated bearing capacity of the specimen can be obtained by using the strength formula of concrete-filled steel tube members [5]. At the beginning of loading stage, The bending moment was applied through jack at the lower part of the specimen, and then the torsional load was applied on specimen progressively. In each loading step, 1/10 of the estimated torsional strength was applied, and the time interval between two continuous loading steps was set as about 2–3 min. When the load was increased to 60 % of the predicted bearing capacity of specimen, continuous load was then employed until the displacement of jack reached limitation. The arrangement of strain flowers is shown in Fig. 3.

### 2.3 Experimental phenomenon

**Fig. 4:**
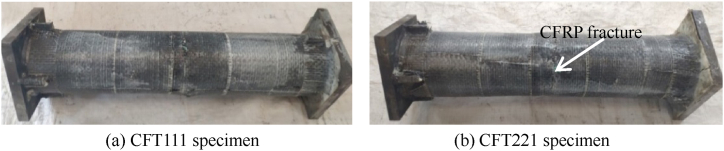
Failure mode of circular concrete filled CFRP steel tube specimen.

At the initial stage of loading, there is no obvious change in each group of specimens, and sporadic adhesive cracking sound can be heard; When the specimen enters the elastic-plastic stage, there is an obvious sound of adhesive cracking. Fig. 4 shows the failure mode of circular concrete filled CFRP steel tube specimen. The specimen with *m* = 0.1 shows obvious torsional deformation, but there is no obvious fracture of CFRP in the mid span of the member, as shown in Fig. 4 (a); The transverse CFRP in the middle of the span of other test pieces is broken to varying degrees and stripped from the steel tube without restraint, as shown in Fig. 4 (b). It can be seen that compared with the torque, the bending moment is the main factor leading to the failure of the CFRP concrete-filled steel tube. The analysis shows that when the member is subjected to the bending moment. The torque only makes the CFRP in the full section tension state, that is, when the specimen is under the bending torsion load, the transverse CFRP fracture degree in the mid span of the member becomes more and more serious.

**Fig. 5:**
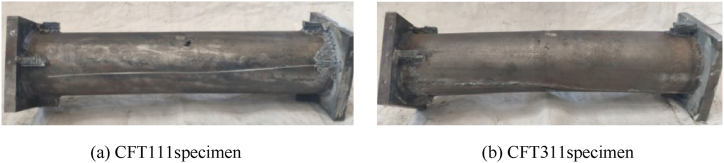
Failure mode of steel tube.

Fig. 5 shows failure mode of steel tube. After the test, after the CFRP of the loaded specimen is cut open, it can be seen that the steel tube of the specimen with relatively small bending moment (*m* = 0.1) is not cracked and has no obvious drum, but has obvious torsional deformation, as shown in Fig. 5 (a). With the increase of bending moment ratio, in addition to torsional deformation, the steel tube also gradually begins to appear bending deformation, and the bending deformation is more significant with the increase of bending moment ratio. As shown in Fig. 5 (b), the bending deformation of steel tube is consistent with the fracture of CFRP, indicating that CFRP and steel tube work well together.

**Fig. 6:**
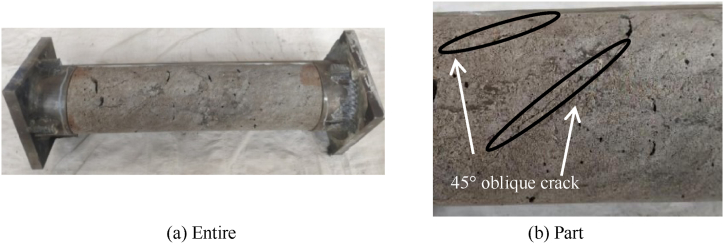
Failure mode of concrete.

Fig. 6 shows failure mode of concrete. The failure morphology of concrete inside the component is shown in Fig. 6(a). When the steel tube is cut open, it can be seen that the internal concrete is intact without crushing and falling off, but its surface is covered with many inclined cracks roughly 45° to the longitudinal axis of the specimen, and most of the cracks are carried out at the bending moment in the middle of the span, as shown in Fig. 6(b). Seel tube and CFRP have good restraint effect on concrete, and concrete mainly suffers torsional failure, which is consistent with the failure mode of steel tube, indicating that concrete and steel tube can ensure good synergy.

### 2.4 Test results analysis

#### 2.4.1 *T*-*θ* curves

The *T*-*θ* curve of all members under bending and torsion load can be divided into three sections.

1) the curve develops linearly.
2) the curve from elastic stage enters the elastic-plastic stage.
3) the later stage of loading enters the plastic stage. Among them, in the later stage of loading, there is no obvious sudden drop in the curve of each specimen.

**Fig. 7:**
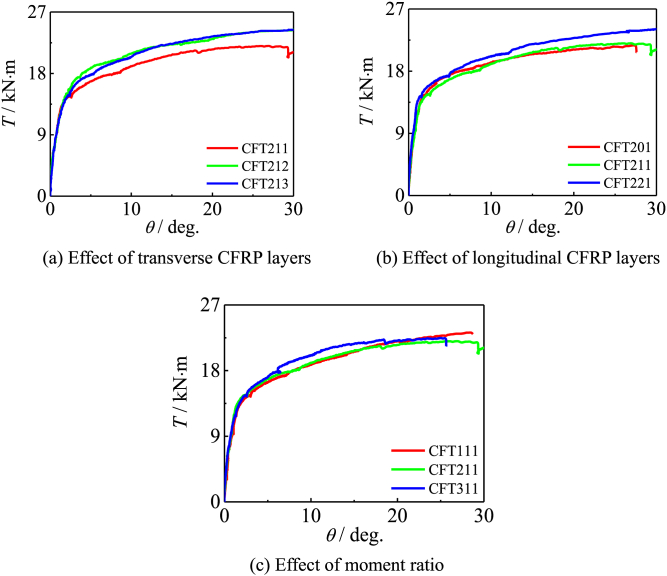
*T*-*θ* curve of specimen.

Fig. 7 shows *T*-*θ* curve of all specimen. The effects of transverse CFRP layers, longitudinal CFRP layers and moment ratio on members are shown in Fig. 7 (a) ∼ (c) respectively. The stiffness in the elastic stage of the curve is related to the cross-sectional area of the material. Since the layer thickness of CFRP is only 0.111 mm, it can be ignored compared with the section size of steel tube and concrete. With the increase of moment ratio, the increase rate of member bearing capacity is faster. When reaching the later stage of loading, the increase of bearing capacity is inversely proportional to the moment ratio. This is because the section displacement in the member increases. Compared with the specimen with small moment ratio, it is necessary to apply a greater torque in the early loading to achieve the same rotation angle; When reaching the later stage of loading, due to the large bending moment applied in the initial stage, the member damage is more serious, and the CFRP cracks in varying degrees.

In order to study the influence of investigated parameters on the mechanical properties of specimens more clearly, bearing capacity and ductility are calculated and shown in Table 5.

**Table 5:** Bearing capacity and ductility of specimens.

Specimens label	Bearing capacity *T*/kN·m	Ratio %	Ductility
CFT111	20.32	100	7.632
CFT211	19.06	93.79	8.19
CFT311	18.93	93.16	8.44
CFT112	20.77	102.21	7.31
CFT212	21.89	107.72	7.16
CFT312	22.04	108.46	6.96
CFT213	21.97	108.12	7.11
CFT201	18.86	92.81	8.32
CFT221	22.06	108.56	6.90

#### 2.4.2 Shear stress-shear strain curve

**Fig. 8:**
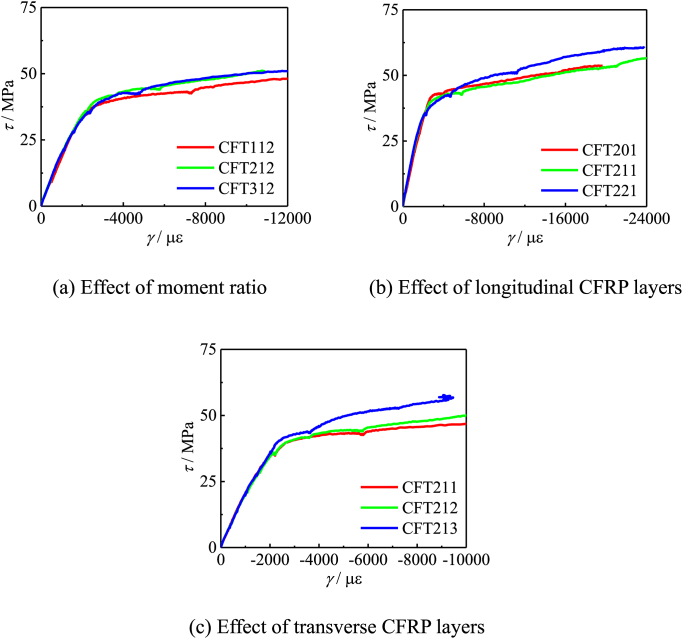
*τ−γ* curve of specimens.

Fig. 8 shows the *τ−γ* curve of specimens. It can be seen from Fig. 8 all *τ−γ* curve can be divided into three stages: elastic stage, elastic-plastic stage, and plastic stage. The effects of transverse CFRP layers, longitudinal CFRP layers and moment ratio on members are shown in Fig. 8 (a) ∼ (c) respectively. With the increase of the number of longitudinal and transverse CFRP layers, the shear stress of the members is increased. The results of the comparison curve show that the bending moment ratio, the number of transverse CFRP layers and the number of longitudinal CFRP layers will not affect the stiffness in the elastic stage, indicating that the shear modulus of these three are not directly related to the strength.

2 Finite element analysis.

### 2.5 Finite element model

The finite element method of specimen is used to simulate the stress-strain relationship of steel tube and concrete. The stress-strain relationship between steel tube and concrete is as follows equation (7)∼(16) [25]:

(7) $$\sigma_{\text{cl}}/\sigma_{0}=\left\{\begin{array}{l} 2\left(\frac{\varepsilon_{\text{cl}}}{\varepsilon_{0}}\right)-{\left(\frac{\varepsilon_{\text{cl}}}{\varepsilon_{0}}\right)}^{2}\left(\varepsilon_{\text{cl}}\leq \varepsilon_{0}\right)\\ \left\{\begin{array}{l} \left[1-q+q{\left(\frac{\varepsilon_{\text{cl}}}{\varepsilon_{0}}\right)}^{0.1\xi }\right]{\left(\frac{\varepsilon_{\text{cl}}}{\varepsilon_{0}}\right)}^{C}\left(\varepsilon_{s}\geq 1.12\right)\\ \frac{{\left(\frac{\varepsilon_{\text{cl}}}{\varepsilon_{0}}\right)}^{1+D}}{\beta {\left(\frac{\varepsilon_{\text{cl}}}{\varepsilon_{0}}-1\right)}^{2}+\frac{\varepsilon_{\text{cl}}}{\varepsilon_{0}}}\left(\varepsilon_{s}<1.12\right) \end{array}\right\}\left(\varepsilon_{0}<\varepsilon_{\text{cl}}\leq \varepsilon_{u}\right)\\ \frac{\frac{\varepsilon_{\text{cl}}}{\varepsilon_{0}}}{\beta_{s}{\left(\frac{\varepsilon_{\text{cl}}}{\varepsilon_{0}}-1\right)}^{2}+\frac{\varepsilon_{\text{cl}}}{\varepsilon_{0}}}\left(\varepsilon_{\text{cl}}>\varepsilon_{u}\right) \end{array}\right\}$$

(8) σ0=f(MPa)c′

(9) ε0=εcc+(600+32.4f)c′ξ0.2×10‐6

(10) $$\varepsilon_{cc}=\left(1300+12.09f_{c}^{\prime }\right)\times 10^{6}$$

(11) $$q=\frac{\xi^{0.745}}{2+\xi }$$

(12) $$C=\zeta^{\prime }\left(2.231-4.611\zeta^{\prime }\right)$$

(13) $$D=\zeta^{\prime }\left(1.545-1.238\zeta^{\prime }\right)$$

(14) $$\beta =3.28{\left(2.36\times 10^{-5}\right)}^{0.25+{\left(\xi -0.5\right)}^{7}}f^{\prime }c^{2}\times 10^{-4}$$

(15) $$\beta_{s}=0.5{\left(2.36\times 10^{-5}\right)}^{0.25+{\left(\xi_{s}-0.5\right)}^{7}}f^{\prime }c^{2}\times 10^{-4}$$

(16) $$\varepsilon_{u}=\varepsilon_{0}+51659\xi_{\text{cf}}‐38904\xi_{\text{cf}}^{2}$$

where: the compressive strength of concrete is defined as *f*'_c_. *ξ*′ Is the hoop coefficient. *q,* C and D are about *ξ* Relevant parameters of. *β* and *β*_*s*_ is about *ξ*_*s*_. *ε*_u_ is the longitudinal strain of the specimen.

For CFRP, transverse CFRP mainly plays a restrictive role, so the limiting factor of transverse CFRP is adopted（*ξ*_cf_) quantification. Longitudinal CFRP plays the role of reinforcement, so its reinforcement efficiency is defined as the reinforcement coefficient (*η*). The calculation of all influencing factors is given by the following formulas(17)∼(19):

(17) $$\eta =\frac{A_{\text{cfl}}f_{\text{cfl}}}{A_{s}f_{y}}$$

(18) $$f_{\text{cft}}=E_{\text{cf}}\varepsilon_{\text{cftr}}$$

(19) $$f_{\text{cfl}}=E_{\text{cf}}\varepsilon_{\text{cflr}}$$

**Fig. 9:**
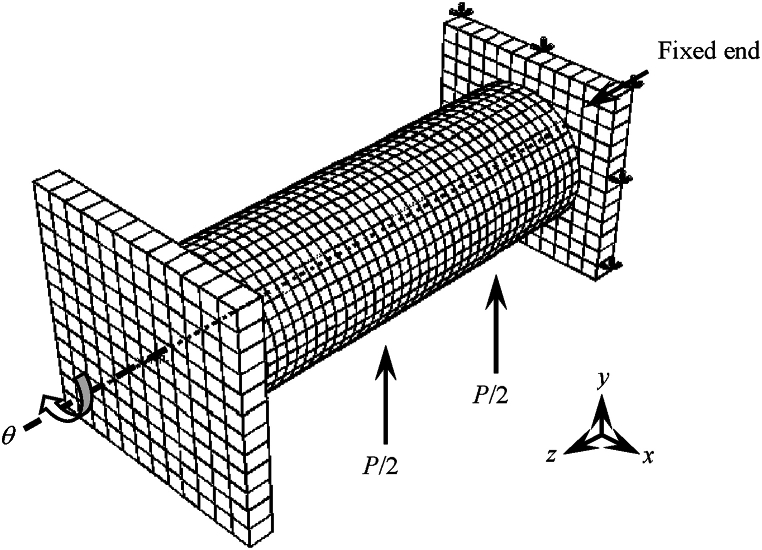
Boundary conditions of FE simulation.

Fig. 9 shows the boundary conditions of finite element simulation of specimen. Boundary conditions refer to Refs. [[16], [17], [18], [19], [20]].

### 2.6 Verification of finite element results

**Fig. 10:**
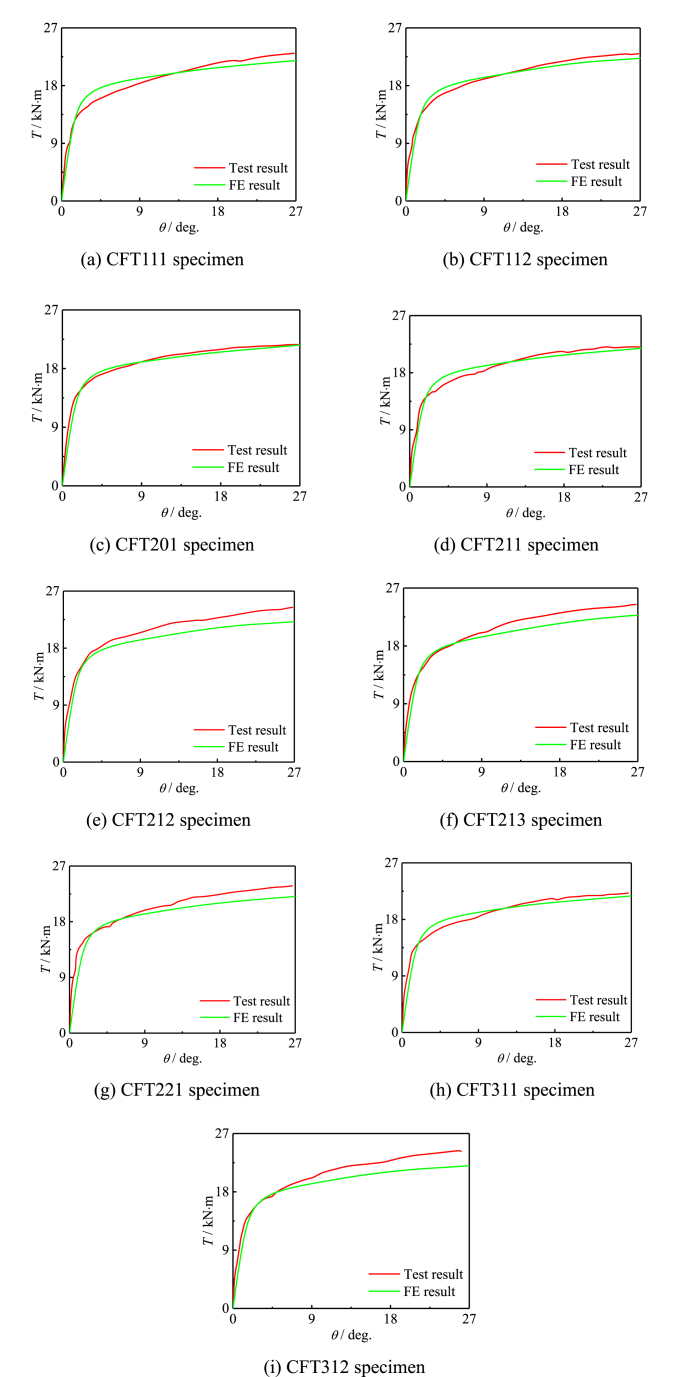
Contract of FEs and test results of specimen.

**Fig. 11:**
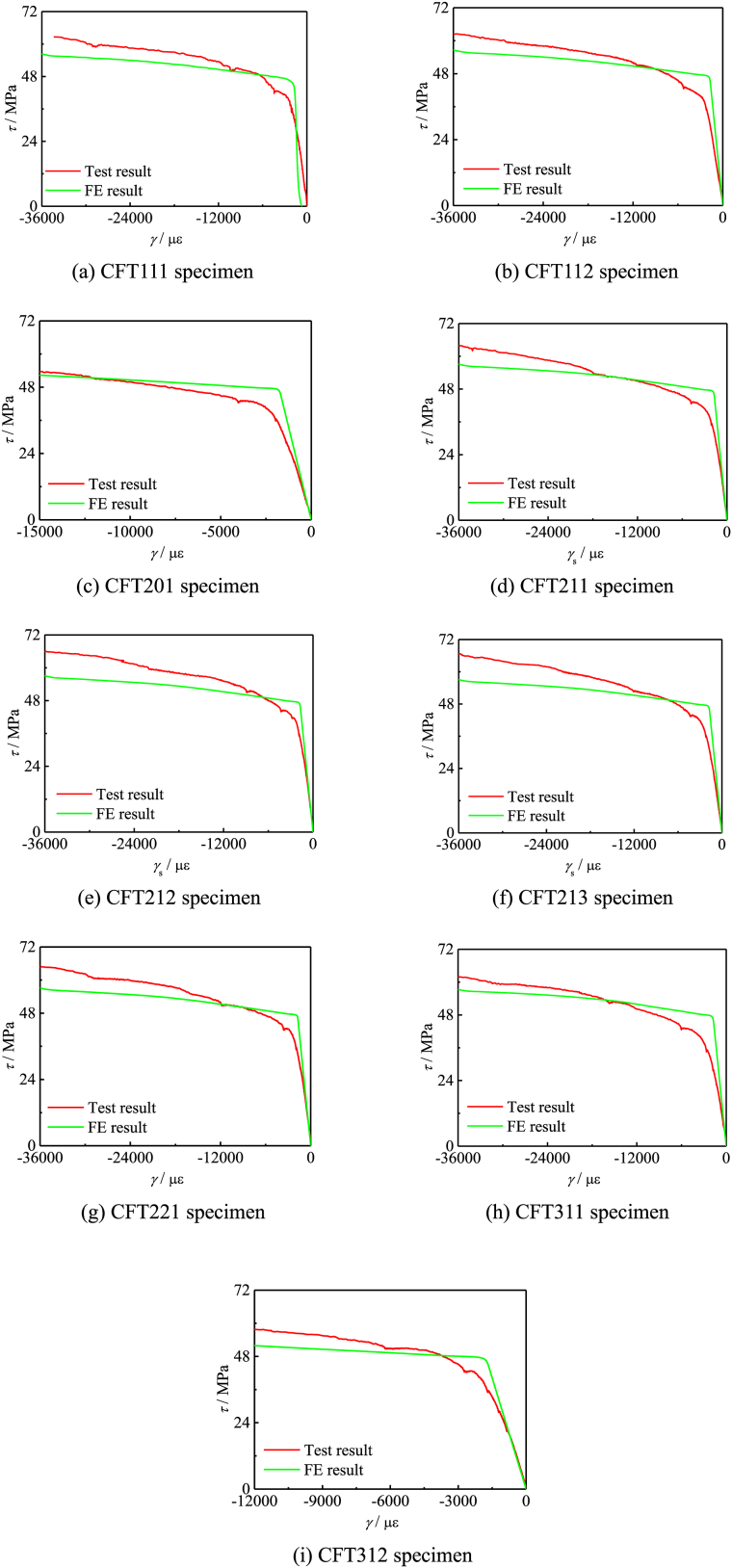
Contract of FEs and test results of specimen.

Fig. 10∼Fig. 11 respectively show comparing situations of the *T*-*θ* curve, *τ−γ* curve. Fig. 10 (a) ∼ (i) shows contract of FEs and test results of *T*-*θ* curve of all specimens, and Fig. 11 (a) ∼ (i) shows contract of FEs and test results of *τ−γ* curve of all specimens. The shear stress-strain curve reflects the change of stress and strain in the member. The average error of 5.71 % and a mean square error of 0.87 for the elastic stage stiffness. The average error of bearing capacity is 4.15 %, with a mean square error of 0.92.

**Fig. 12:**
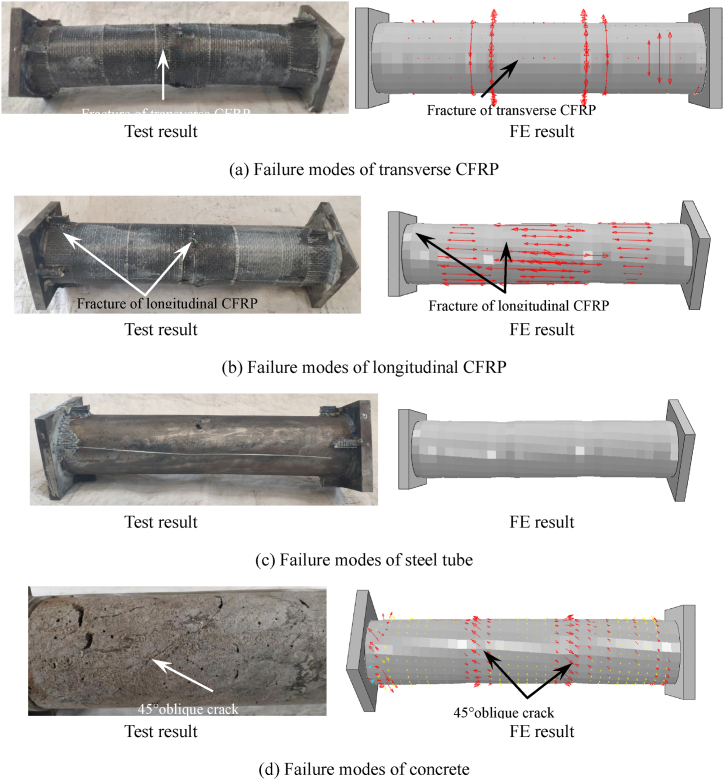
Contract of FEs and test results of specimen.

Fig. 12 respectively show comparing situations of fail mode. Fig. 12 (a) ∼ (d) shows the comparison between the simulation results and test results of the failure modes of circular CFRP concrete filled steel tubular specimens, transverse CFRP, longitudinal CFRP, steel tube and internal concrete. Fig. 12 (a) ∼ (b) shows the comparison between the CFRP test and the simulated failure mode of the specimen. It can be seen that in the finite element simulation, the arrows representing the stress in the transverse CFRP mid span and both sides of the longitudinal CFRP disappear, indicating that the CFRP in this part of the model has failed and broken. Fig. 12 (c) shows the comparison between steel tube test and simulated failure mode. It can be seen that the simulated failure mode of steel tube is in good agreement with the test. Fig. 12 (d) shows the concrete failure mode of specimen. In the simulation results, inclined cracks with an angle of 45° appear in the core concrete.

## 3 Analysis of the whole process of stress

**Fig. 13:**
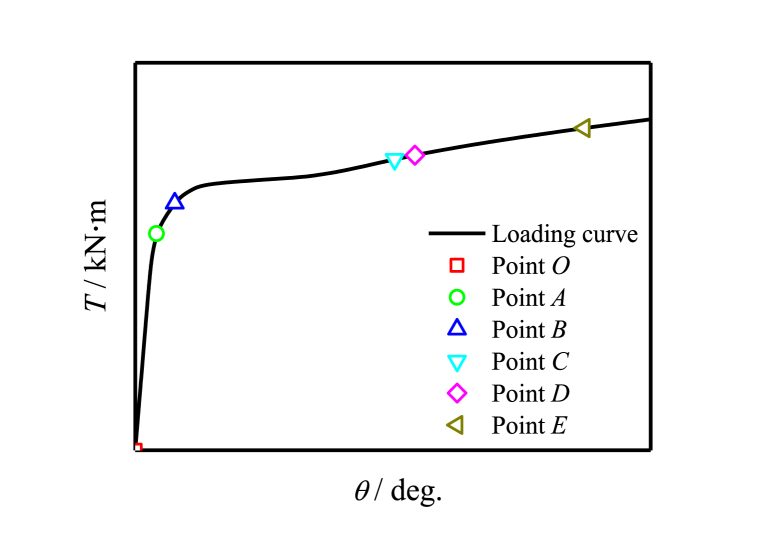
Typical *T-θ* curve of specimen.

Fig. 13 shows the typical *T*-*θ* curve of members.

### 3.1 Distribution of all materials

**Fig. 14:**
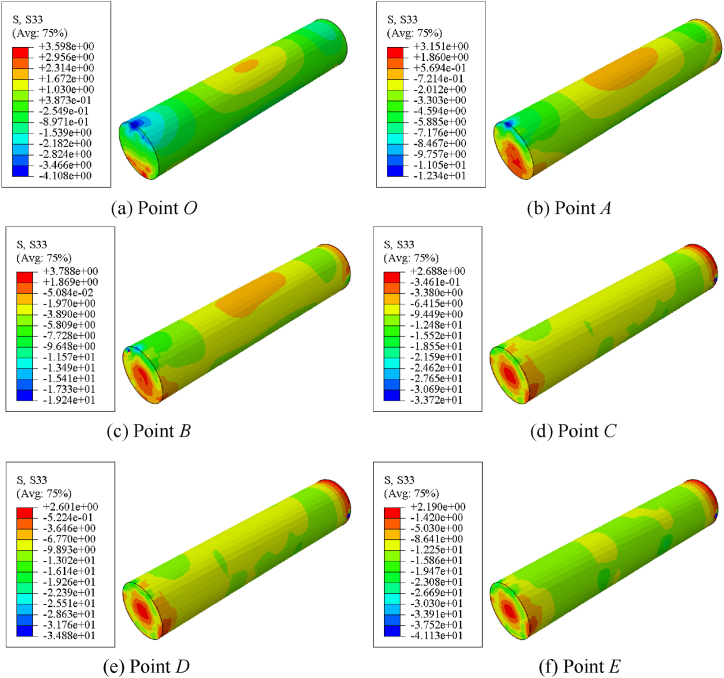
Longitudinal stress distribution of specimen.

Fig. 14 shows the longitudinal stress distribution of specimen. Fig. 14 (a) ∼ (f) shows the stress distribution of concrete with different characteristic points. After point B, Stress redistribution and gradual increase in stress at both ends.

**Fig. 15:**
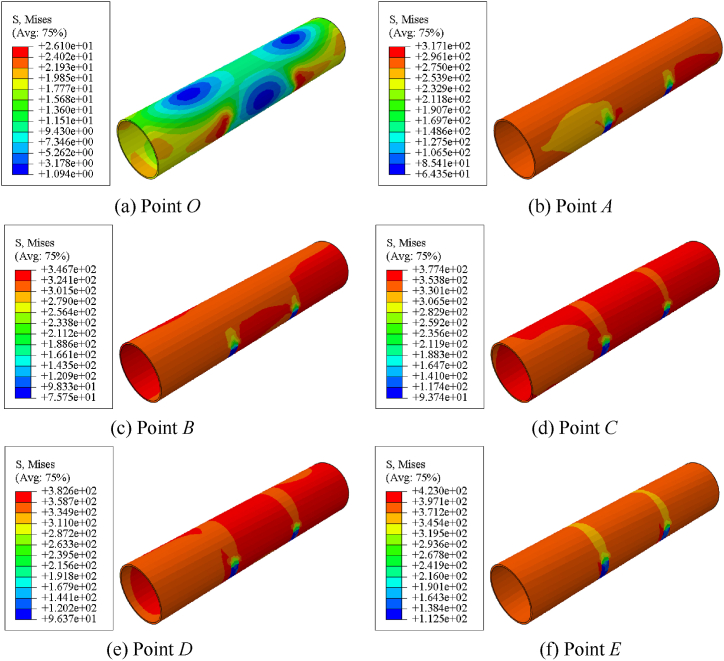
Mises stress distribution of specimen.

Fig. 15 shows the Mises stress distribution of member. Fig. 15 (a) ∼ (f) shows stress distribution of steel with different characteristic points. When the steel is loaded to point B or even point C, the stress of the steel tube increases greatly from elasticity to yield, and the stress reaches 345 MPa; After point D, the steel enters the strengthening stage, and the macroscopic manifestation is an increase in stress value, which is significantly higher than the yield stress.

### 3.2 Parameter analysis

**Fig. 16:**
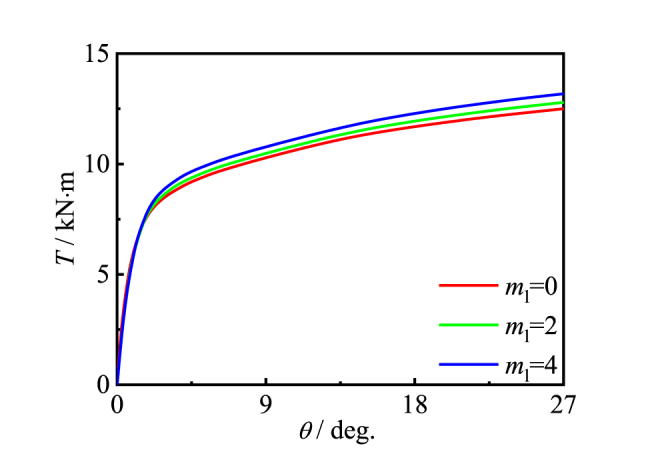
Effect of *m*_l_ on *T*-*θ* curve of member.

**Fig. 17:**
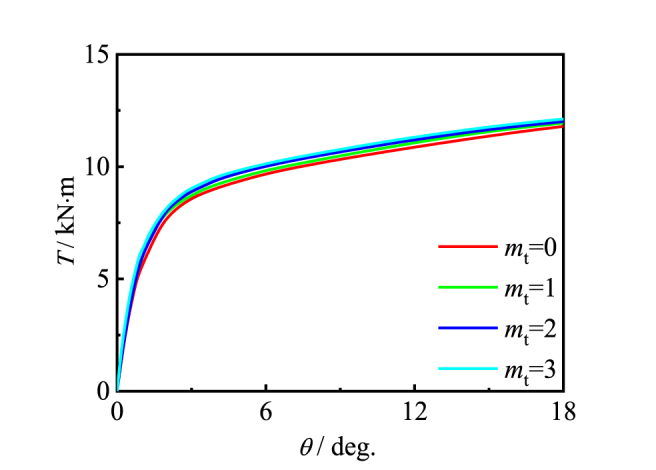
Effect of *m*_t_ on *T*-*θ* curve of member.

Fig. 16, Fig. 17 is effects of the number of longitudinal and transverse CFRP layers. Due to the good restraint effect, the good encapsulation results in an slightly increase in the bearing capacity of the specimen with m_t_/m_l_ increases.

**Fig. 18:**
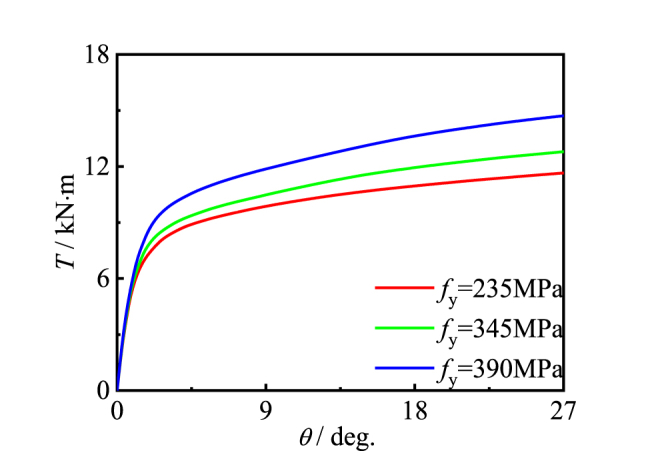
Effect of *f*_y_ on *T*-*θ* curve of member.

**Fig. 19:**
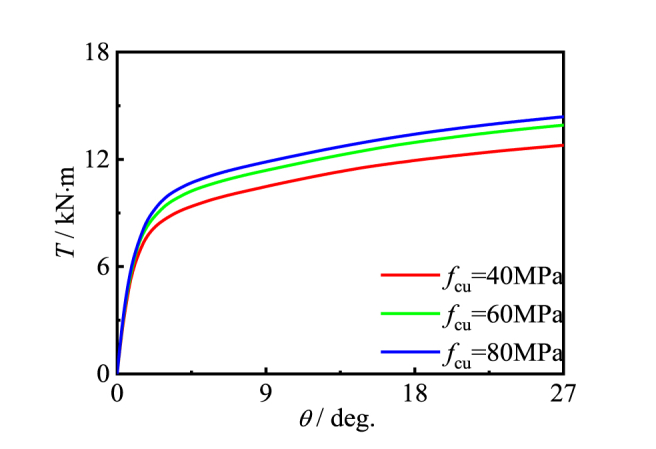
Effect of *f*_cu_ on *T*-*θ* curve of member.

Fig. 18, Fig. 19 show the effects of property on members. With the change of the initial stiffness and the shape of the material, the bearing capacity increases obviously. This is because steel tube and concrete are the main components of CFRP concrete-filled steel tube members, and their material properties have an important impact on the bearing capacity. With the increase of steel yield strength and concrete compressive strength, the overall bearing capacity of members has been significantly improved.

**Fig. 20:**
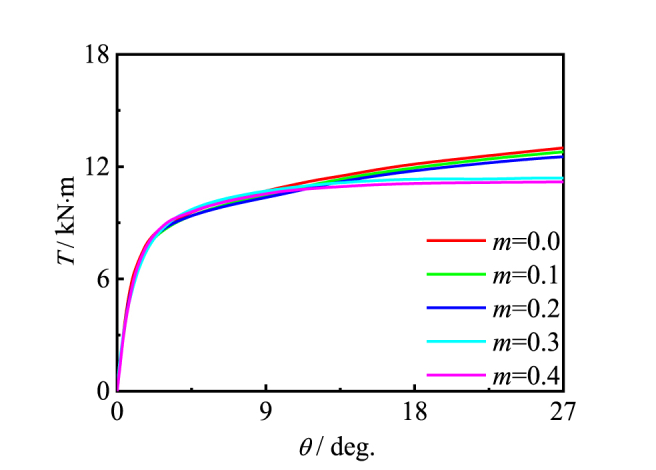
Effect of *m* on *T*-*θ* curve of member.

Fig. 20 shows the effect of the bending moment ratio on the performance of the specimen. Because the moment ratio has a great influence on the loading process of the specimen, bearing capacity decreases slightly.

**Fig. 21:**
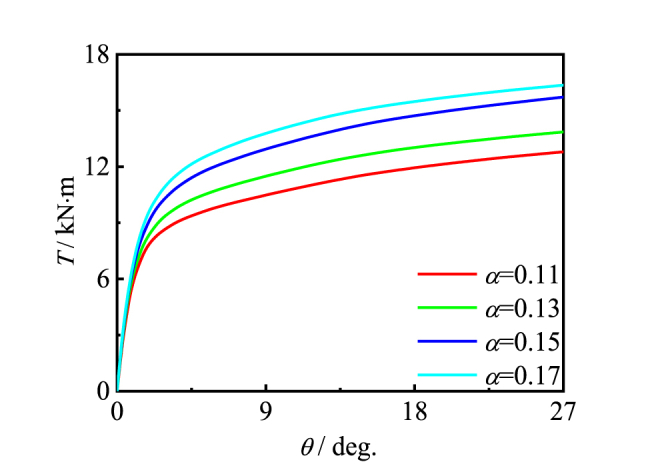
Effect of *α* on *T*-*θ* curve of member.

Fig. 21 shows the effect of steel content (*α*) on the member. Steel content and capacity are positively correlated. The analysis shows that the steel content has a great impact on the overall bearing capacity of circular members. This is because the increase of steel content leads to the increase of carbon content of steel, which significantly improves the hardness, strength and brittleness of steel [26].

## 4 Bearing capacity correlation equation

### 4.1 Correlation equation

**Fig. 22:**
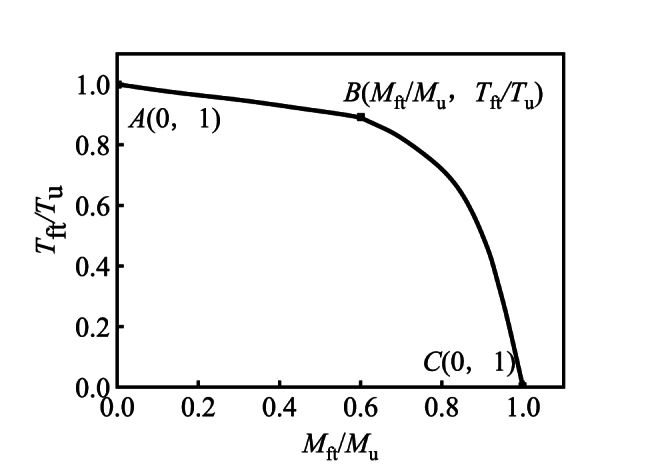
Typical *M*_ft_/*M*_u_*-T*_ft_/*T*_u_ curve of specimen.

Fig. 22 shows the typical *M*_ft_/*M*_u_-*T*_ft_/*T*_u_ curve of flexural torsional members.

Through the calculation results of finite element method, the stress curve of bending torsion member is fitted. According to the test phenomenon and the change of bearing capacity, the strain in the tensile zone of the bending torsion member is defined as *ε*_max_, which corresponds to the torsional bearing capacity

(20) $$\varepsilon_{\max}=2837+166800/D_{s}$$

The expression is as follows.

(1) *A*-*B* stage（0.1≤*M*_ft_/*M*_u_ < 0.5）

(21) $$T_{\text{ft}}/T_{u}+aM_{\text{ft}}/M_{u}=1$$

(2) *B*–*C* stage（0.5≤*M*/*M*_u_ ≤ 0.9）

(22) $$‐{\left(M_{\text{ft}}/M_{u}\right)}^{2}+bM_{\text{ft}}/M_{u}+T_{\text{ft}}/T_{u}=1$$

where：*a* = 0.983、*b* = 0.7218。

### 4.2 Validation of expressions

**Fig. 23:**
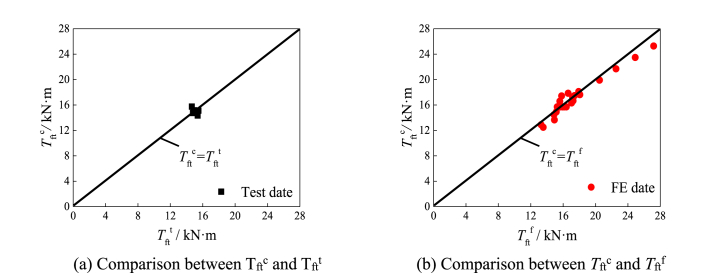
Comparison of verification results of specimen.

Fig. 23 shows comparison of verification results of specimen. Fig. 23 (a) and Fig. 23 (b) shows comparison of verification results of specimen. Average value of component *T*_ft_^c^/*T*_ft_^t^ is 0.998 and the mean square deviation is 0.038; The mean value of *T*_ft_^c^/*T*_ft_^f^ is 0.973 and the mean square deviation is 0.051. It can be seen that the results calculated by the bearing capacity equation are in good agreement with the test results and finite element results.

## 5 Conclusion

(1) The torsional load-displacement curve of all specimens can be approximately divided into elastic stage, elasto-plastic stage, and descending stage. The specimens' CFRP can work together with CFST. Through calculation, the ductility of the specimen is good. The bearing capacity of the specimen is directly proportional to the number of CFRP layers and inversely proportional to the bending moment ratio.
(2) The torsional load of all specimens is relatively close. Although experiencing almost the same stress process, the strength reserve of specimen is sufficient.
(3) Simulated failure mode of steel tube and concrete are in good agreement with the test.
(4) Through calculation, it is found that the average value of component Tftc/Tftt is 0.998 and the mean square deviation is 0.038; The average value of Tftc/Tftf is 0.973 and the mean square deviation is 0.051, which shows that the results calculated by the bearing capacity equation are in good agreement with the test results and finite element results.
(5) Coupling effects of compression-bending-torsional loads of concrete filled CFRP steel tube would be conducted research on the that are closer to actual working conditions in the future.

## Funding

This research was funded by Project for Talent of Liaoning Province of China grant number No. XLYC1902009, the Talent Program of Chengdu Technological University (2023RC041), and the Laboratory Open Fund of Chengdu Technological University.

## Data availability

Data associated with our study has not been deposited into a publicly available repository. The datasets will be made available on request.

## CRediT authorship contribution statement

**Qing-li Wang:** Conceptualization. **Hai-yu Qin:** Formal analysis. **Kuan Peng:** Resources.

## Declaration of competing interest

No potential conflict of interest was reported by the authors.

## Nomenclature

*A*_c_: Cross-sectional area of concrete
*A*_cfl_: Cross-sectional area of longitudinal CFRP
*A*_cft_: Cross-sectional area of transverse CFRP
*A*_s_: Cross-sectional area of steel tube
*B*: Side-width of steel tube
CF-CFRP-ST: Concrete filled CFRP-steel tubes
CF-CFRP-T: Concrete filled CFRP tube
CFRP: Carbon fiber reinforced plastic
CFST: Concrete filled steel tube
CFT: Concrete filled tube
*E*_c_: Elastic modulus of concrete
*E*_cf_: Elastic modulus of carbon fiber sheet
*E*_s_: Elastic modulus of steel tube
*f*_cfl_: Ultimate tensile strength of longitudinal CFRP
*f*_cft_: Ultimate tensile strength of transverse CFRP
*f*'_cf_: Tensile strength of carbon fiber sheet
*f*_ck_: Characteristic compressive strength of cubic concrete
*f*_cu_: Compressive strength of cubic concrete
*f*_u_: Ultimate tensile strength of steel tube
*f*_y_: Yield strength of steel tube
*L*: Length of specimen
*m*_l_: Number of layer(s) of longitudinal CFRP
*m*_t_: Number of layer(s) of transverse CFRP
S–CF–CFRP-ST: Square concrete filled CFRP-steel tube
S-CFST: Square concrete filled steel tube
*T W*_cfscm_: Torque:Flexural modulus of the member
*t*_cf_: Thickness of one-layer carbon fiber sheet
*t*_s_: Wall-thickness of steel tube
*v*_s_: Poisson's ratio of steel tube
*ε*: Strain
*ε*_45_: Strain in 45^o^ direction
*ε*_cf_: Strain of CFRP
*ε*': Elongation percentage of steel tube
*ε*_cf45_: Strain of CFRP in 45^o^ direction
*ε*_cfl_: Strain of Longitudinal CFRP
*ε*_cflr_: Rupture strain of longitudinal CFRP
*ε*_cft_: Strain of transverse CFRP
*ε*_cftr_: Rupture strain of transverse CFRP
*ε*_l_: Longitudinal strain
*ε*_s_: Strain of steel tube
*ε*_s45_: Strain in 45^o^ direction of steel tube
*ε*_sl_: Longitudinal strain of steel tube
*ε*_st_: Transverse strain of steel tube
*ε*_t_: Transverse strain
*γ*: Shear strain
*η*_cf_: Strengthening factor of longitudinal CFRP
*θ*: Rotation angle
*ξ*_cf_: Confinement factor of transverse CFRP
*ξ*_s_: Confinement factor of steel tube
